# Clinical validation of time reduction strategy in continuous step-and-shoot mode during SPECT acquisition

**DOI:** 10.1186/s40658-021-00354-x

**Published:** 2021-02-02

**Authors:** Valentin Picone, Nikolaos Makris, Fanny Boutevin, Sarah Roy, Margot Playe, Michael Soussan

**Affiliations:** 1 GE Healthcare, 78530, 283 Rue de la Miniere, Buc, France; 2 Department of Nuclear Medicine, Avicenne Hospital, HUPSSD, APHP, Paris, France; 3 Inserm, Institut Curie, Laboratoire d’Imagerie Translationnelle en Oncologie, Orsay, France

**Keywords:** SPECT/CT, SwiftScan, Lungs, Bone, SUV, Semi-quantification

## Abstract

**Background:**

The SwiftScan solution (*General Electric Healthcare*) combines a new low-energy high-resolution sensitivity collimator and a tomographic step-and-shoot continuous (SSC) mode acquisition. The purpose of this study is to determine whether SSC mode can be used in clinical practice with shorter examination times, while preserving image quality and ensuring accurate semi-quantification. Twenty bone scan and 10 lung scan studies were randomly selected over a period of 2 months. Three sets of image datasets were produced: step-and-shoot (SS) acquisition, simulated 25% count reduction using the Poisson resampling method (SimSS), and SimSS continuous acquisition (SimSSC), where SimSS was summed with counts acquired during detector head rotation. Visual assessment (5-point Likert scale, 2 readers) and semi-quantitative evaluation (50 focal uptake from 10 bone studies), assessed by SUV_mean_, coefficient of variation (COV), and contrast-to-noise ratio (CNR), were performed using *t* test and Bland-Altman analysis.

**Results:**

Intra-reader agreement was substantial for reader 1 (*k* = 0.71) and for reader 2 (*k* = 0.61). Inter-reader agreement was substantial for SS set (*k* = 0.93) and moderate for SimSSC (*k* = 0.52). Bland-Altman analysis showed a good interchangeability of SS and SimSSC SUV values. The mean CNR between SS and SimSSC was not significantly different: 42.9 ± 43.7 [23.7–62.1] vs. 43.1 ± 46 [22.9–63.3] (*p* = 0.46), respectively. COV values, assessing noise level, did not deviate significantly between SS and SimSSC: 0.20 ± 0.08 [0.18–0.23] vs. 0.21 ± 0.08, [0.18–0.23] (*p* = 0.15), respectively, whereas a significant difference was demonstrated between SS and SimSS: 0.20 ± 0.08 [0.18–0.23] vs. 0.23 ± 0.09 [0.20–0.25] (*p* < 0.0001), respectively.

**Conclusions:**

SSC mode acquisition decreases examination time by approximately 25% in bone and lung SPECT/CT studies compared to SS mode (~ 2 min per single-bed SPECT), without compromising image quality and signal quantification. This SPECT sensitivity improvement also offers the prospect of more comfortable exams, with less motion artifacts, especially in painful or dyspneic patients.

## Background

**Fig. 1:**
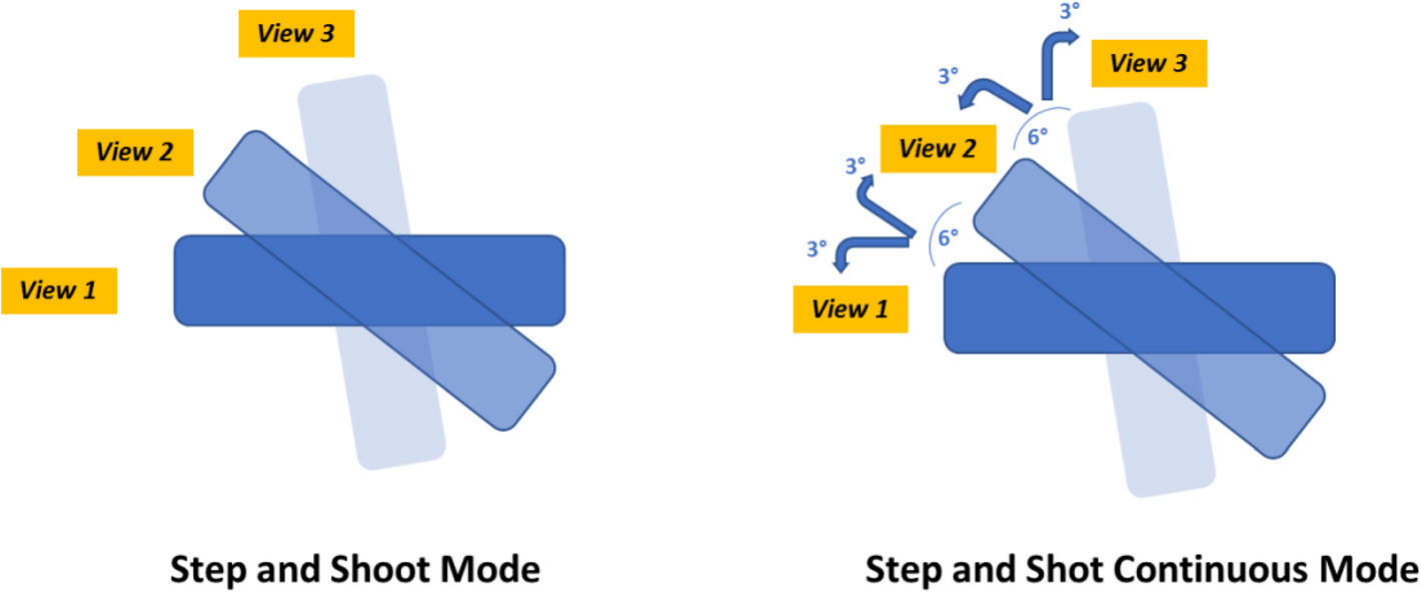
An illustration of step-and-shoot (SS) versus step-and-shoot continuous (SSC) mode acquisition, demonstrating incorporation of counts during the detector head rotation in SSC mode

Over the last years, several technological advances have been integrated to single-photon emission computed tomography (SPECT) [1]. The introduction of computed tomography (CT)-based attenuation correction, scatter correction, and resolution recovery, as well as the introduction of new cadmium zinc telluride (CZT) detectors, has allowed physical phenomena that occur along the pathway of the photon until its detection to be taken into account, rendering semi-quantification in SPECT/CT attainable [2–4]. Besides, different SPECT image acquisition strategies have also been suggested in the nuclear medicine practice. Three modes of SPECT acquisition are available: step-and-shoot (SS), continuous, and step-and-shoot continuous (SSC). In the SS mode, projection data are acquired only when the detector is stationary, whereas in the SSC mode, data are acquired both when the detector is stationary and when the detector moves from one view to the next [5]. During the SSC mode, the system acquires events from three locations: the regular SS position, the half arc immediately preceding the SS position, and the half arc immediately following it. The counts acquired during the 6° rotation are partitioned as follows: between 0° and 3°, counts are assigned in position 0°, and between 3° and 6°, they are integrated in the next position 6° (Fig. 1).

Although this is a relatively long-standing concept introduced in the mid-90s by Cao et al. [5], who studied the effect in image quality using a SSC versus a conventional SS mode acquisition using computer simulations, only recently was SSC evaluated in a clinical setting. A recent paper of a French team [6] observed on phantoms that SSC mode improves volumetric sensitivity compared to SS mode, without significant impact on image quality. These results suggest that a strategy of time or dose reduction could be applied in a clinical setting.

The SwiftScan solution (General Electric Healthcare) combines a new low-energy high-resolution sensitivity collimator (LEHRS) and a tomographic SSC mode acquisition. The purpose of this study is to determine whether SSC mode can be used in clinical practice with shorter examination times, while preserving image quality and ensuring accurate semi-quantification.

## Methods

### Patient study

Twenty patients with 20 bone scan studies and 10 patients with lung scan studies were randomly selected over a period of 2 months between July and November 2018 (Table 1). All studies were performed using a SPECT/CT D870DR gamma camera (GE Healthcare, Waukesha, WI). This gamma camera is coupled to a 16-slice CT scanner and includes latest technological advancements, on iterative reconstruction and metal artifact correction. Bone scans were performed using a double bed position, covering dorsal and lumbar spine, following an administration of 727 ± 82 MBq of ^99m^Tc-osteocis. For lung scans, the average dose of Technegas® administered to the patients was 407 ± 40 MBq (ventilation) and 193 ± 9 MBq of ^99m^Tc-labeled albumin macro-aggregates (perfusion).

**Table 1:** Characteristics of the patients

**Patients with bone scans (*n* = 20)**	
Weight (kg, mean ± SD)	70,6 ± 13,7
Osteocis® administered activity (MBq, mean ± SD)	727 ± 82
Time to acquisition (minutes, mean ± SD)	228 ± 47
Indications	
Bone metastatic disease assessment	7/20
Bone pain/arthralgias	6/20
Suspicion of prosthesis loosening or infection	5/20
Suspicion of complex regional pain syndrome	2/20
**Patients with lung scans (*n* = 10)**	
Administered activity (MBq, mean ± SD)	
Technegas® (ventilation)	407 ± 40
Pulmocis® (perfusion)	193 ± 9
Indications	
Suspected pulmonary embolism	9/10
Pre-operative evaluation	1/10

### Image acquisition and reconstruction protocols

The SPECT acquisition protocols were performed with a two-head camera, with 60 projections over 360°, 180° per detector, step of 6°, 15 and 16 s/projection for bone and lung studies, respectively.

All images were reconstructed on a *Xeleris 4 DR* workstation with the following parameters:

For bone studies: ordered subsets expectation maximization reconstruction (OSEM) algorithm with 6 subsets, 3 iterations, and a Gaussian filter (FWHM = 5.3 mm). Each bone image was corrected for attenuation, scatter, and resolution recovery based on collimator-detector pair response.

For lung studies: OSEM algorithm with 2 iterations, 8 subsets, and a Butterworth filter with *f*_*cut*_ = 0.6 cycle/cm and *p* = 5. We intended to only visually assess these lung cases; thus, no quantitative corrections were applied.

In order to simulate a decrease in time reduction, low count statistic images were reconstructed with a 25% time reduction in acquisition using the raw data from the SS mode and using a Poisson resampling algorithm [7]. The detector rotation time from projection *N* to projection *N* + 1 is in the order of approximately 2 s (~ 2 min per single-bed SPECT study). As this study contains bone and lung protocols with time per projection of about 16 s, the selection of 25% time reduction will even out the addition of counts during head rotation between projections. Lower or higher time per projection acquisitions may slightly increase or decrease, respectively, the projected time gain. Poisson sampling is a process in which each element of the population is subjected to an independent Bernoulli test that determines whether the element becomes part of the sample. Each element of the population may have a different probability of being included in it. This probability when drawing a single sample is identified by the first order inclusion probability of that element. If all first order inclusion probabilities are equal, Poisson sampling becomes equivalent to Bernoulli sampling, which can therefore be considered as a particular case of Poisson sampling. Mathematically, the first-order inclusion probability of the second element of the population is designated by the symbol π_i_. The probability of second order inclusion, when a couple consisting of the *i*th and *j*th elements of the population sampled, is included in a sample during the formation of a single sample rated π_ij_. The following relationship is valid during Poisson sampling: *π*_*ij*_ = *π*_*i*_ × *π*_*j*_

### Data analysis

In this study, three set of image datasets were produced as follows (Table 2):

- Set 1. Step-and-shoot (SS) acquisition
- Set 2. Simulated SS (SimSS): simulated 25% count-reduction using the Poisson resampling method
- Set 3. Simulated SS and continuous (SimSSC) acquisition: set 2 was summed with counts acquired during detector head rotation.

**Table 2:** Time parameters for three acquisition modes in bone and lung SPECT/CT protocols

Acquisition mode/organ	Bone SPECT	Lung SPECT
Step-and-shoot (SS)	16 s	15 s
Simulated step-and-shoot (− 25%) (SimSS)	12 s	11 s
Simulated step-and-shoot (− 25%) continuous (SimSSC)	12 s + C	11 s + C

*C:* counts acquired during detector rotation between projections

In this study, only set 1 and set 3 are used for both visual assessment and semi-quantitative evaluation. Set 2 was produced as an intermediate dataset to create set 3.

### Visual assessment

The 30 SPECT studies were visually evaluated by two experienced nuclear medicine physician (reader 1: M.S. and reader 2: M.P.). The reviewers compared blindly SS and SimSSC images and attributed a grade using a 5-point ordinal scale (Likert score) [8] to evaluate the image quality based on the following criteria: non-diagnostic image quality/resolution (grade 0), sub-optimal diagnostic and limited clinical information (grade 1), diagnostic and acceptable image quality/resolution (grade 2), diagnostic and good image quality/resolution (grade 3), and diagnostic and excellent image quality/resolution (grade 4).

### Semi-quantitative evaluation

For the semi-quantitative evaluation, only bone studies were analyzed. Five lesions were segmented per patient, making a total of 50 volume of interests (VOIs). We chose to use the mean standardized uptake value (SUV_mean_) instead of SUV_max_ to avoid the influence of elevated noise in the measurement. The volumes employed as well as their localization, in all three series, were identical. The SUV_mean_ was calculated by first selecting the coronal slice with the highest uptake, for each hot spot, in the conventional set of images. Subsequently, a SUV threshold-based segmentation was initiated by placing a seed point on the lesion and the contour was progressively adapted to the lesion boundaries until the threshold criteria were fulfilled. SPECT and CT images were used to employ in a muscle region (e.g., quadriceps) a threshold-based background VOI, encompassing a volume of 10–20 cc. For assessing image *noise* level, standard deviation of SUV values within each lesion and background VOI was used to calculate the coefficient-of-variation (COV), which was given by the following equation:

$$ \mathrm{COV}=\frac{{\mathrm{SD}}_{\mathrm{SUV}\mathrm{mean}}}{{\mathrm{SUV}}_{\mathrm{mean}}} $$

where SUV_mean_ and SD_SUVmean_ correspond to the average and standard deviation of within lesion and background VOIs.

The contrast to noise ratio (CNR) was also calculated and was given by the following equation:

$$ \mathrm{CNR}=\frac{N_{\mathrm{lesion}}-{N}_{\mathrm{background}}}{\sigma_{\mathrm{background}}} $$

where *N*_lesion_ and *N*_background_ correspond to the mean activity concentration of the VOI_lesion_ and the VOI_background_, respectively, and *σ*_background_ is the standard deviation of the VOI_background_. The VOI_background_ was selected in the muscle. All SUV analysis was performed with *Q.Volumetrix MI* (GE Healthcare, Milwaukee) on a Xeleris 4 DR workstation.

### Statistical analysis

Data are presented as mean ± standard deviation (SD) [95% confidence interval]. To assess the consistency of visual assessments, the Cohen’s kappa (*k*) [9] for intra-reader and inter-reader agreements was calculated for all scans. Similarity of homogeneity of variance between the quantitative variables of the 3 sets was verified using the Leven’s test. The Bland-Altman method [10] was used for comparing SUV_mean_ and SD_SUVmean_ between SS and SimSSC sets. Mean and SD were reported as well as lower and upper limits of agreement (LOA), calculated as ± 1.96 × SD. A Student *t* test was used to test the null hypothesis at the 0.05 significance level, along with 95% confidence intervals (95% CI); for comparison of the SUV, COV, and CNR between the 3 sets, statistical tests were performed using Excel spreadsheets.

## Results

### Visual comparison between SS and SimSSC images

**Fig. 2:**
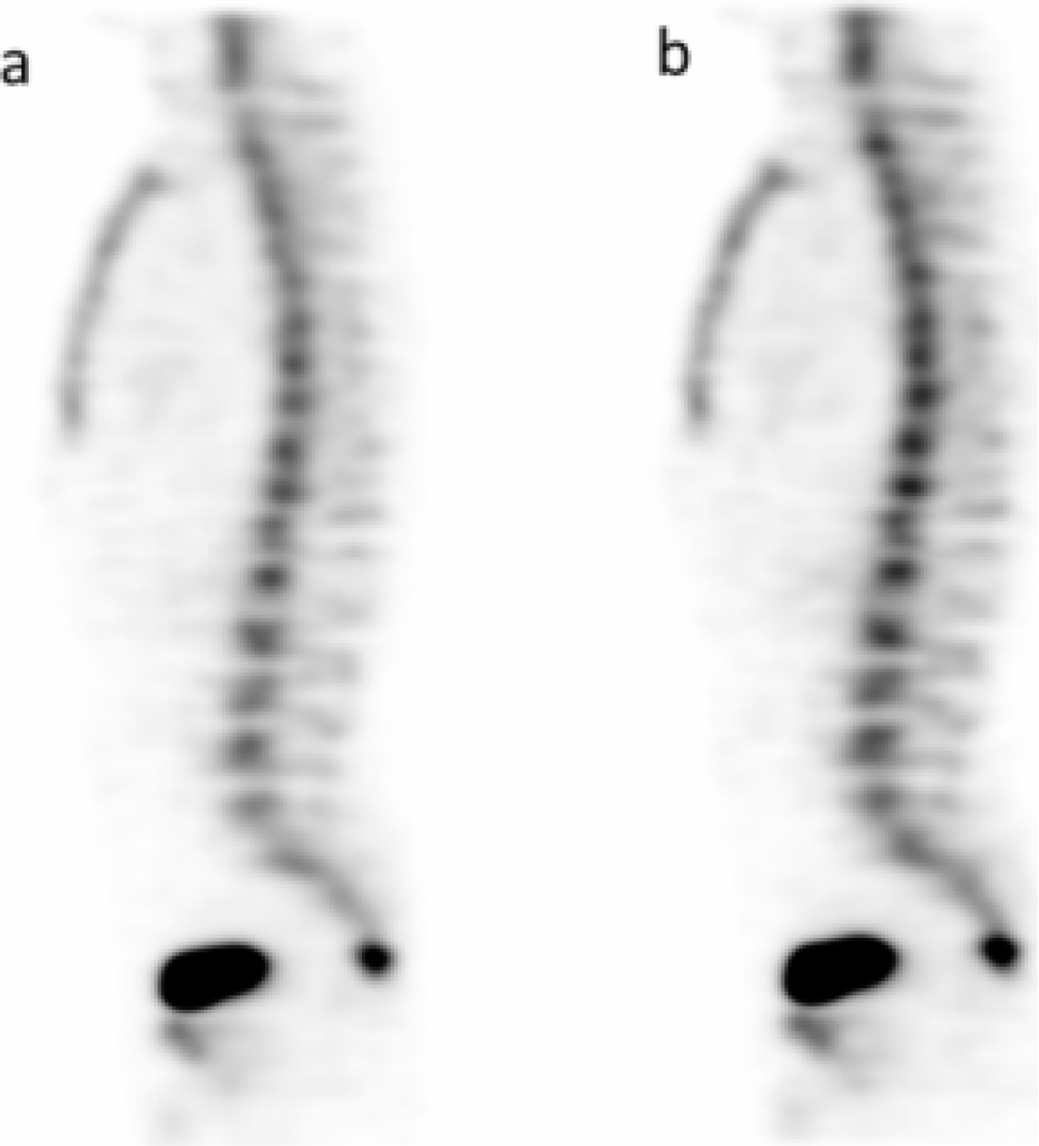
Bone SPECT. Comparison between SS (**a**) and SimSSC (**b**) images on a sagittal view. Images were rated as 4 for the 2 modes

**Fig. 3:**
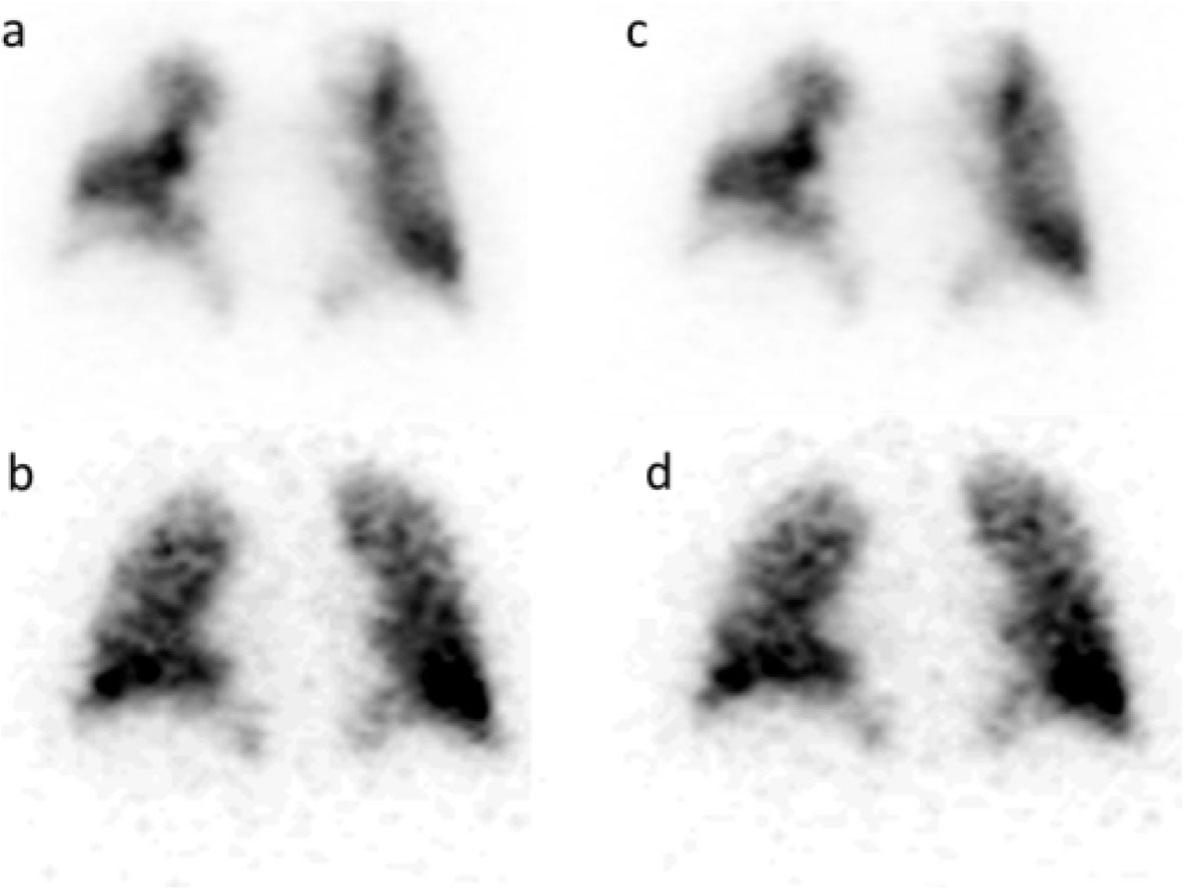
V/Q lung SPECT. Comparison between SS (**a** perfusion; **b** ventilation) and SimSSC (**c** perfusion; **d** ventilation) images on coronal views. Images were rated as 4 for the 2 modes

Image quality was graded as 3/4 in 90% (18/20) of bone studies and 90% (9/10) of lung studies for both readers. Intra-reader agreement (between grades 3 and 4) was substantial for reader 1 (*k* = 0.71) and for reader 2 (*k* = 0.61). Inter-reader agreement was substantial for SS set (*k* = 0.93) and moderate for SimSSC (*k* = 0.52). Visual examples of image quality of bone and lung studies are shown in Figs. 2 and 3.

### Semi-quantitative comparison between SS and SimSSC images

**Fig. 4:**
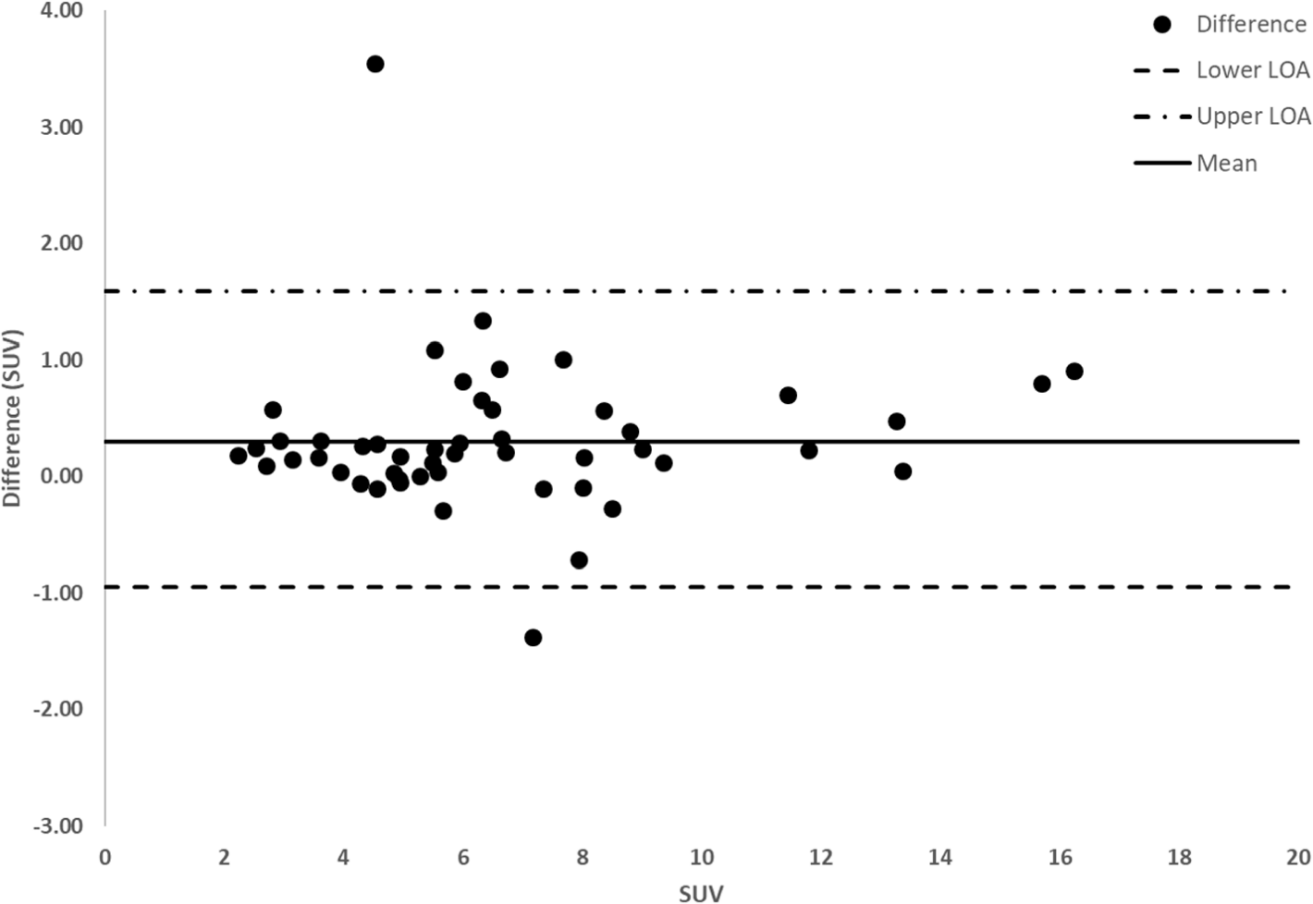
Bland-Altman plot demonstrating differences in SUV as a function of average SUV between step-and-shoot (SS) and simulated step-and-shoot continuous (SimSSC) mode, pooling 50 lesions from 10 patients (5 lesions per patient)

**Fig. 5:**
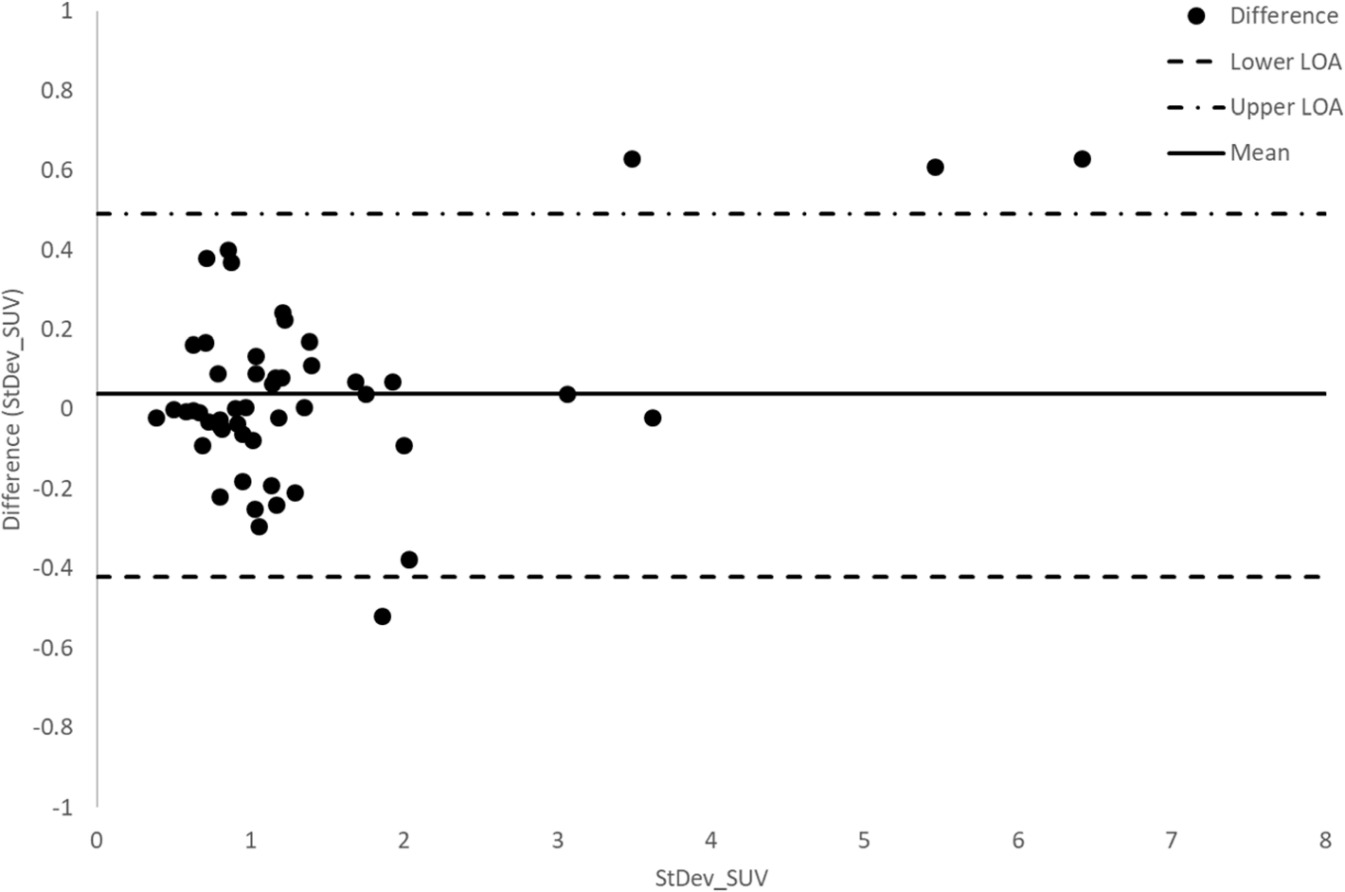
Bland-Altman plot demonstrating differences in standard deviation of SUV as a function of averaged standard deviation SUV between step-and-shoot (SS) and simulated step-and-shoot continuous mode (SimSSC), pooling 50 lesions from 10 patients

Semi-quantitative comparison between SS and SimSSC of the 50 VOIs was assessed by means of SUV, COV, and CNR. SUV values were not significantly different between SS and SimSSC: 6.8 ± 3.3 [0.4–13.2] vs. 6.5 ± 3.2 [0.2–12.8] (*p* = 0.6), as well as SD_SUVmean_ values: 1.4 ± 1.2 [− 1–3.8] vs. 1.4 ± 1.1 [− 0.8–3.6] (*p* = 0.9). A graphical representation of the data using Bland-Altman plots is shown between SS and SimSSC sets for SUV_mean_ in Fig. 4 and for SD_SUVmean_ in Fig. 5. The mean difference of SS-SimSSC was 0.3 ± 0.6 [− 1–1.6] for SUV_mean_ and 0.04 ± 0.23 [− 0.4–0.5] for SD_SUVmean_, while pooling fifty bone lesions across 10 patients.

**Fig. 6:**
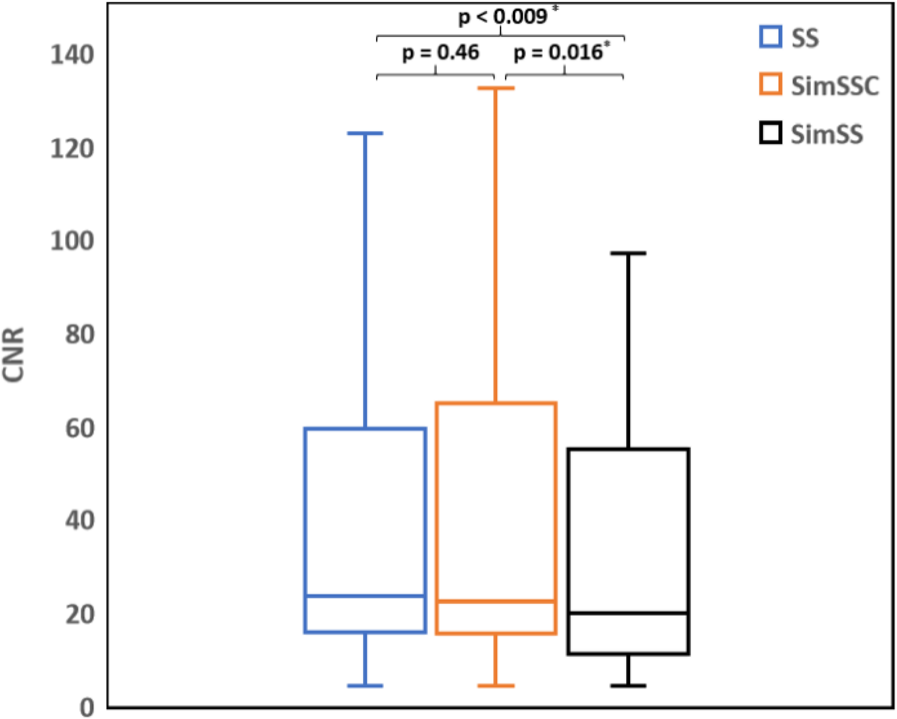
Box plot on contrast-to-noise ratio for two acquisition modes (SS, step-and-shoot; SimSSC, simulated step-and-shoot continuous; SimSS, simulated step-and-shoot). The median is illustrated by the midline, first and third quartiles by the lower and upper lines of the box, and extremes by whiskers

In Fig. 6, the use of SimSSC resulted in similar CNR mean values as compared with SS mode: 43.1 ± 46 [95% CI: 22.9–63.3] vs. 42.9 ± 43.7 [23.7–62.1] (*p* = 0.46) whereas CNR values with SimSS mode (31.7 ± 27.1 [19.8–43.6]) differed significantly from SS (*p* = 0.009) and SimSSC (*p* = 0.016).

**Fig. 7:**
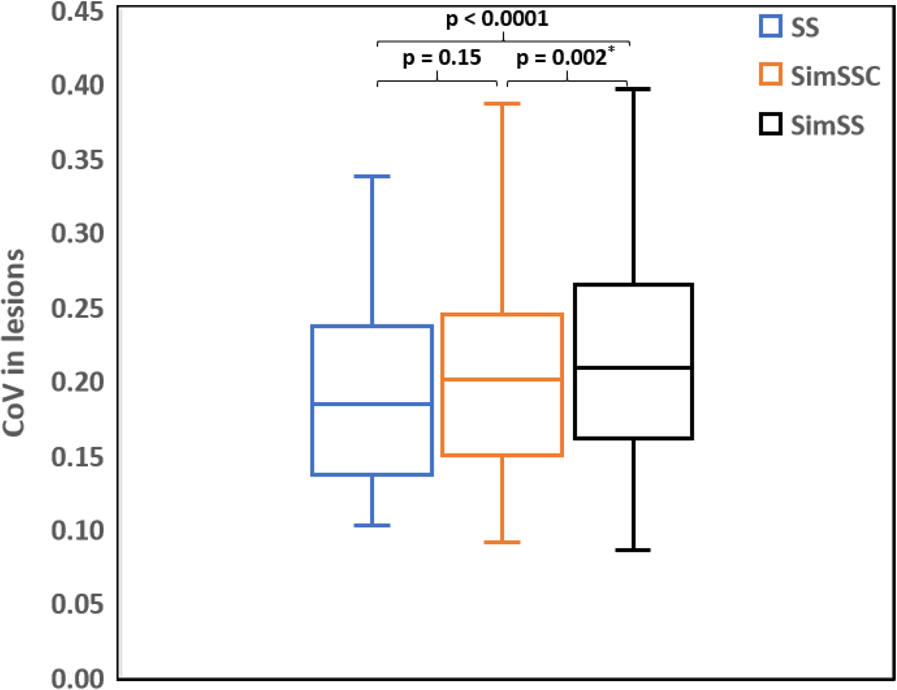
Box plot showing the coefficient-of-variation of the 50 lesions, for three acquisition modes (SS, step-and-shoot; SimSSC, simulated step-and-shoot continuous; SimSS, simulated step-and-shoot). The median is illustrated by the midline, first and third quartiles by the lower and upper lines of the box, and extremes by whiskers

Figure 7 shows the average COV obtained using the three acquisition modes. COV mean values did not deviate significantly between SS and SimSSC: 0.20 ± 0.08 [95% CI: 0.18–0.23] vs. 0.21 ± 0.08 [0.18–0.23]; (*p* = 0.15), whereas a significant difference was demonstrated between SS and SimSS: 0.20 ± 0.08 [0.18–0.23] vs. 0.23 ± 0.09 [0.20–0.25] (*p* < 0.0001).

## Discussion

The use of SPECT has been significantly increased these last years. Many strategies have been developed with the aim of reduction of injected activity as well as the time procedure, aiming at improving patient experience and reducing anxiety prior to and during SPECT imaging [11]. Nuclear cardiac imaging benefits the most of these strategies, especially with the introduction of dedicated cardiac cameras [12]. However, in the field of extra cardiac imaging, such as bone or lung studies, there is still a need for improvement in SPECT time duration without compromising image quality and quantification.

In this paper, we studied the clinical validity on visual and semi-quantitative assessment of a time reduction strategy during SPECT acquisition using SSC mode acquisition. In general, we demonstrated that incorporation of counts during detector rotation between projections with a 25% decrease of scan time (~ 2 min per single-bed SPECT study) provides an equivalent image quality and accurate quantitative values with respect to standard SS acquisition. In the same way, the SSC mode can also compensate for an equivalent of 25% dose reduction.

Bland-Altman analysis shows that the vast majority of the SUV values is around the mean line of the differences between SS and SimSSC (+ 0.3) and almost always between the LOA (except 2 outliers). There is also a small clinically insignificant bias (+ 0.3) between SS and SimSSC. The difference between LOA is indeed of the order of 2.6 SUV units, which represents about 38% of the average of SUVs (6.8). The topic of SUV variability in PET imaging has been extensively covered the past decade demonstrating that changes in the acquisition, reconstruction, and post-processing parameters may introduce significant variability in SUV measurements. In clinical practice, if the comparison is made with PET, a threshold of at least 30% of SUV variation is considered significant and attributable to a biological effect, while also ensuring a meaningful comparability of the two acquisitions. In our study, the difference is of the same order, which points to a non-significant clinical impact of the variability of the measurement between SS and SimSSC. In addition, the use of SUV ratio is also common in SPECT quantification which allows to cancel out plasma clearance and/or cross calibration related differences [13].

These observations show the interchangeability of SUV measurements of SS and SimSSC, as no significant bias was observed, and noise level was not increased in SimSSC.

For both bone and lung studies, visual assessment confirmed the similarity in image quality between SS (bone 16 s/step; lung perfusion 15 s/step) and SimSSC images (bone 12 s/step; lung perfusion 11 s/step; lung ventilation 15 s/step). The noise introduced into the image by the decrease of the counting statistics is well compensated by the activation of the continuous mode. This is corroborated by an excellent COV correlation and no CNR significant differences between SS and SimSSC whereas SimSS demonstrated, as expected, significant different COV values to SS due to the simulated reduction of exam duration. The image quality of bone and lung studies is therefore maintained qualitatively and semi-quantitatively while reducing time examination by 25% (from 15 s to 11 s for lung perfusion and from 16 s to 12 s for bone).

Acquiring counts during head rotation is a rather new concept; therefore, only a few publications have been so far appeared in scientific literature. A recent study validated the effects of detector rotation speed and rotation time for continuous repetitive rotation acquisition on image quality and quantification in DaTSCAN SPECT. It was demonstrated that a combination of rotation speed and rotation times affect the image quality and quantification of DaTSCAN SPECT [14]. Overall, the authors suggested the use of added projection data processes and proper rotation speed when continuous repetitive rotation acquisition is applied. In another study by Bailly et al. [13], it was demonstrated that Swiftscan step and shoot continuous acquisition may enable a 25% time reduction of DaTSCAN acquisitions without changing visual and/or semi-quantitative analysis while reporting on striatal binding SUV ratios. These findings are in agreement with those reported in our study, thus confirming the utility of incorporating additional projections in a SPECT acquisition or demonstrating the possibility for reduced acquisition times without affecting image quality.

Semi quantitative analysis is ready to become a standard in SPECT studies [4, 15]. The integration of corrections in SPECT imaging such as for photon attenuation and scatter, the use of semi-automatic delineation of volumes of interest and normalization factors (body weight and injected dose) renders semi-quantitative comparison of activity concentrations possible, allowing to reduce SUV variability in follow-up studies. As there is a growing need in SPECT imaging to obtain activity concentrations, this study suggests that SUV quantification and image properties are not altered with the SSC acquisition mode, and therefore, bone and lung SPECT studies can even be performed with shorter duration.

Beyond the gain in scan time or dose reduction, the clinical impact of using SSC instead of conventional SS mode is worth to be evaluated, especially in terms of quantification accuracy, lesion detectability, and characterization.

## Conclusions

Step-and-shoot continuous mode acquisition decreases examination time by approximately 25% in bone and lung SPECT studies compared to step-and-shoot mode (~ 2 min per single-bed SPECT study), without compromising image quality and signal quantification. This SPECT sensitivity improvement also offers the perspective of more comfortable exams, with less motion artifacts, especially in painful or dyspneic patients

## Data Availability

The datasets used and/or analyzed during the current study are available from the corresponding author on reasonable request.

## Abbreviations

CNR: Contrast-to-noise ratio
COV: Coefficient-of-variation
LOA: Limit of agreement
OSEM: Ordered subsets expectation maximization reconstruction
SimSS: Simulated step-and-shoot
SimSSC: Simulated step-and-shoot continuous
SPECT/CT: Single-photon emission computed tomography/computed tomography
SS: Step and shoot
SSC: Step-and-shoot continuous
SUV: Standardized uptake value
VOI: Volume of interest
